# Antipyretic Therapy in Critically Ill Patients with Established Sepsis: A Trial Sequential Analysis

**DOI:** 10.1371/journal.pone.0117279

**Published:** 2015-02-24

**Authors:** Zhongheng Zhang

**Affiliations:** Department of critical care medicine, Jinhua municipal central hospital, Jinhua hospital of Zhejiang university, Zhejiang, P.R.China; Mario Negri Institute for Pharmacology Research, ITALY

## Abstract

**Background and objective:**

antipyretic therapy for patients with sepsis has long been debated. The present study aimed to explore the beneficial effect of antipyretic therapy for ICU patients with sepsis.

**Design:**

systematic review and trial sequential analysis of randomized controlled trials.

**Database:**

Pubmed, Scopus, EBSCO and EMBASE were searched from inception to August 5, 2014.

**Methods:**

Mortality was dichotomized as binary outcome variable and odds ratio (OR) was chosen to be the summary statistic. Pooled OR was calculated by using DerSimonian and Laird method. Statistical heterogeneity was assessed by using the statistic I^2^. Trial sequential analysis was performed to account for the small number of trials and patients.

**Main results:**

A total of 6 randomized controlled trials including 819 patients were included into final analysis. Overall, there was no beneficial effect of antipyretic therapy on mortality risk in patients with established sepsis (OR: 1.02, 95% CI: 0.50–2.05). The required information size (IS) was 2582 and our analysis has not yet reached half of the IS. The Z-curve did not cross the O’Brien-Fleming α-spending boundary or reach the futility, indicating that the non-significant result was probably due to lack of statistical power.

**Conclusion:**

our study fails to identify any beneficial effect of antipyretic therapy on ICU patients with established diagnosis of sepsis. Due to limited number of total participants, more studies are needed to make a conclusive and reliable analysis.

## Introduction

Fever is commonly seen in intensive care unit (ICU) patients and is often managed with antipyretics and external cooling.[1] Two primary causes of fever involve central nervous system and infection. The former is associated with the impairment of the central temperature regulation system (e.g. as those in patients with disorders of central nervous system or brain injury), and the latter involves inflammatory response to infectious agents. While therapeutic hypothermia has been proven to be beneficial for those caused by central nervous system, there is a great deal of controversy in the later condition.

Sepsis is defined as the systematic inflammatory response syndrome (SIRS) caused by suspected or documented infections, and it is often accompanied with fever. The management of fever induced by sepsis varies substantially across different institutions and hospitals.[2,3] Some clinicians believe that fever is potentially harmful because it increases oxygen consumption and further exacerbates the imbalance between oxygen supply and demand.[4] Therefore, they employ both antipyretic drugs and external cooling for fever control. With respect to the choice of methods for fever control, some investigators suggest that fever control with antipyretic drugs is potentially harmful because of the side effect of these drugs (e.g. hepatotoxicity of NSAIDS), and external cooling is preferable.[5] However, other investigators think that fever is a protective response elicited by human body to combat microorganisms and suppression of fever may delay the recovery.[6,7]Therefore they do not recommend routine use of fever control strategy in patients with sepsis.

There is no consensus on the management of fever in patients with sepsis. One systematic review has focused on the effect of antipyretic therapy on mortality in febrile ICU patients and the result was neutral.[8] The study population in that study is febrile ICU patients and the result may not be extrapolated to septic patients. Furthermore, new evidence has been published since that systematic review. Therefore, we conducted the systematic review and meta-analysis to investigate the effect of antipyretic therapy on mortality in patients with sepsis.

## Methods

### Study selection

Electronic databases including Pubmed, Scopus, EBSCO and EMBASE were searched from inception to August 5, 2014. The core search consisted terms related to sepsis and antipyretic therapy (“sepsis” or “septic” or “infection” AND “antipyretic” or “antipyretics” or “cooling” or “hypothermia”). The detailed searching strategy and the number of citations obtained from each database are listed in the appendix file (S1 Searching). There was no language restriction. References of reviews and relevant observational studies were searched by hand to identify potential relevant RCTs.

Studies to be included should meet the following criteria: 1) randomized controlled trial investigating the efficacy of antipyretic therapy; 2) study population was adult; 3) the medical condition included sepsis, severe sepsis or septic shock. Definition of sepsis should be explicitly made by the combination of SIRS and infection.[9,10] Studies investigating hypothermia in brain injury were excluded.

### Data extraction

Data on the following information were extracted from included RCTs: the name of the first author, publication year, study population (sepsis, severe sepsis or septic shock), illness severity (reported by severity score such as APACHEII or SAPS), intervention (antipyretic medication or physical cooling), treatment period (how long did the intervention last), sample size, definition of mortality (the definition for primary outcome was chosen if a study reported more than one mortality), other study endpoints. For the calculation of pooled effect size, the numbers of subjects and events (e.g. death) in each arm were extracted.

### Assessment of risk of bias

Risk of bias of each component studies were assessed by using Cochrane Collaboration Risk of Bias Tool.[11] The tool consisted of seven aspects corresponding to different components of RCT design.

### Data synthesis

Mortality was dichotomized as binary outcome variable and odds ratio (OR) was chosen to be the summary statistic. Pooled OR was calculated by using a weighted average of the intervention effects estimated from individual studies. Because component studies included different septic patients in terms of illness severity, causes of sepsis, and infection site, we assumed that different studies estimated different, yet related, intervention effects. We performed random-effects meta-analysis by using DerSimonian and Laird method.[12] Statistical heterogeneity was assessed by using the statistic I^2^, which describes the percentage of the variability in effect estimates that is due to heterogeneity rather than sampling error (chance). An I^2^<30% indicates that the heterogeneity may not be important, and an I^2^>75% indicates considerable heterogeneity, and a value in between indicate moderate heterogeneity.[13]

### Trial sequential analysis

Because meta-analysis containing a small number trials and a small number of patients is subject to spurious findings, we performed trial sequential analysis to adjust for the threshold for which results were considered statistically significant.[14] Information size was calculated by making the following assumptions: relative risk reduction was 20%; mortality rate in control arm was 30%; and the statistical power was 75%. Due to the heterogeneity of included trials, heterogeneity-adjustment factor was employed for information size calculation. O’Brien-Fleming α–spending function is used to adjust for Z-values, because it produced conservative boundaries at early stage where only a small amount of data has been collected.[15] Futility testing is performed by using β-spending function to explore whether the non-significance is due to lack of power or due to underlying equivalence between two arms.

### Reporting bias

Publication bias was assessed by using Egger’s regression test, in which the standard normal deviate (the odds ratio divided by its standard error) is regressed against the estimates precision.[16] The intercept of the regression line is an estimate of asymmetry: the larger its deviation from origin the more significant asymmetry. Funnel asymmetry may be caused by publication bias or small study effect.

### Subgroup analysis

It is plausible that intervention effect may be more pronounced in more severely ill patients, therefore subgroup analysis was performed by restricting subjects to septic shock. Treatment time period as defined by the time in which study protocol was performed was another potential determinant of effect size, thus subgroup analysis was performed by treatment time>48 or <48 hours. Subgroups using different antipyretic treatment (antipyretic medication vs. external cooling) were analyzed.

## Results

### Searching results and component trials

**Fig 1:**
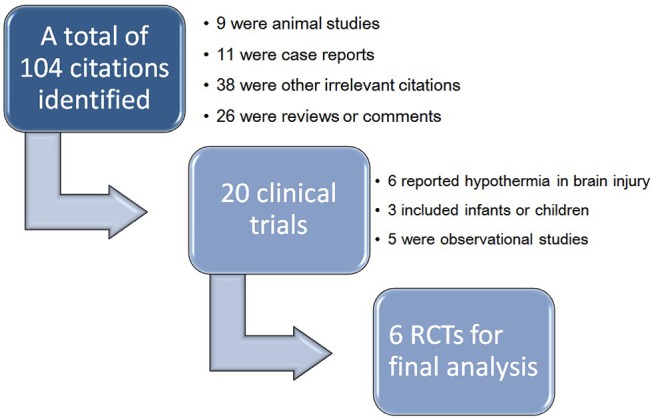
Flowchart of study selection. The initial search identified 402 citations after exclusion of duplicates. 382 of them were excluded by reviewing the titles and abstracts. The remaining 20 articles were clinical studies, in which six studies investigated hypothermia in patients with brain injury, three involved infants or children, and five were observational studies. As a result, a total of 6 randomized controlled trials were included into final analysis.

The initial search identified 104 citations and 84 of them were excluded by reviewing the titles and abstracts. The remaining 20 articles were clinical studies, in which six studies investigated hypothermia in patients with brain injury, three involved infants or children, and five were observational studies. As a result, a total of 6 randomized controlled trials were included into final analysis (Fig. 1).[17–22] Three studies[17–19] included patients with sepsis, one[20] included severe sepsis and the remaining two[21,22] included septic shock. Studies included sepsis and septic shock did not mean that they excluded patients with septic shock but the term “sepsis” covered severe sepsis and septic shock. Therefore, the proportions of septic shock were reported for four studies.[17–20] Severity of patients was presented by using APACHE score or SAPS-3. Three studies[17–19] used ibuprofen as the antipyretic medication and one[20] used lornoxicam for antipyretic therapy. The remaining two studies[21,22] employed external cooling. The study protocol lasted for less than 48 hours in four studies[17–19,21] and for 72 hours in the remaining two studies[20,22]. With respect to definition of mortality, two studies[17,19] used 30-day mortality. Other secondary outcomes were various including shock reversal, serum concentrations of ibuprofen, organ failure, duration of mechanical ventilation, vasopressor use and tissue perfusion parameters (Table 1).

**Table 1:** Characteristics of included RCT studies.

**Studies**	**Study population**	**Shock (%; intervention vs control)†**	**Severity of illness†**	**Intervention**	**Treatment period**	**Sample size**	**Mortality**	**Other study endpoint**
Bernard GR 1991[17]	Sepsis	50 vs 36	-	Ibuprofen	12 hours	30	30-day	Shock reversal; ibuprofen level
Haupt MT 1991[18]	Sepsis	50 vs 31	-	Ibuprofen	22 hours	29	-	Concentration of ibuprofen
Bernard GR 1997[19]	Sepsis	65 vs 63	APACHEII: 16 vs 15	Ibuprofen	48 hours	455	30-day	Organ-failure
Memiş D 2004[20]	Severe sepsis	35 vs 40	APACHEII: 17.1 vs 18	lornoxicam	72 hours	40	Hospital mortality	Mechanical ventilation
Schortgen F 2012[21]	Septic shock	All	SAPS-3: 77 vs 79	External cooling	48 hours	200	14-day;	Vasopressor use; ICU mortality; hospital mortality
Yang YL 2013[22]	Septic shock	All	APACHEII: 23.8 vs 22	External cooling	72 hours	65	28-day	Tissue perfusion

Abbreviations: APACHEII, Acute Physiology and Chronic Health Evaluation II; ICU, intensive care unit; SAPS, Simplified Acute Physiology Score.

† Mean values were reported for the treatment and control arms, respectively

### Assessment of risk of bias

Risks of bias were assessed using seven items corresponding to essential aspects of study design (Table 2). Random sequence generation was rated as low risk of bias in five studies[17–21] except for the study Yang YL 2013[22]. The allocation concealment was not explicitly reported in five studies[17–20,22]. Blinding of participants and outcome assessor was thought of low risk of bias because mortality was unlikely to be distorted by subjective sense. There was no selective reporting in most studies and no other risk of bias was thought to be present.

**Table 2:** Assessment of risk of bias†.

**Studies**	**Random sequence generation**	**Allocation concealment**	**Blinding of participants**	**Blinding of outcome assessor**	**Incomplete outcome data**	**Selective reporting**	**Other bias**
Bernard GR 1991[17]	Low	Unclear	Low	Low	Low	Low	Low
Haupt MT 1991[18]	Low	Unclear	Low	Low	Low	Low	Low
Bernard GR 1997[19]	Low	Unclear	Low	Low	Low	Low	Low
Memiş D 2004[20]	Low	Unclear	Low	Low	Low	Unclear	Low
Schortgen F 2012[21]	Low	Low	Low	Low	Low	Low	Low
Yang YL 2013[22]	Unclear	Unclear	Low	Low	Low	Low	Low

† Risk of bias were assessed according to the handbook of Cochrane. Each item was graded as “low”, “high” and “unclear”.

### Evidence synthesis

**Fig 2:**
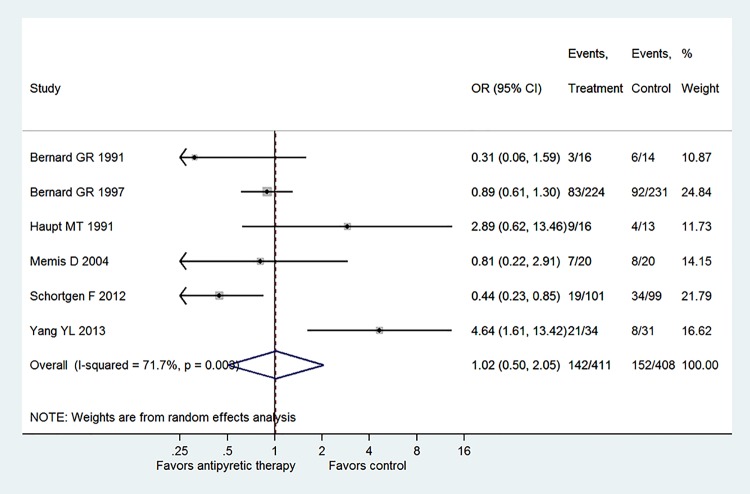
Overall effect pooled across all included trials by using random-effects model. Only one study showed statistically beneficial effect of antipyretic therapy on mortality risk (OR: 0.44, 95% CI: 0.23–0.85), and other trials all showed neutral effect. There was significant heterogeneity among component trials as indicated by an I^2^ of 71.7% (p = 0.003). A total of 819 patients were included in the meta-analysis, including 411 in the treatment group and 408 in the control group. Overall, there was no beneficial effect of antipyretic therapy on mortality risk in patients with established sepsis (OR: 1.02, 95% CI: 0.50–2.05).

**Fig 3:**
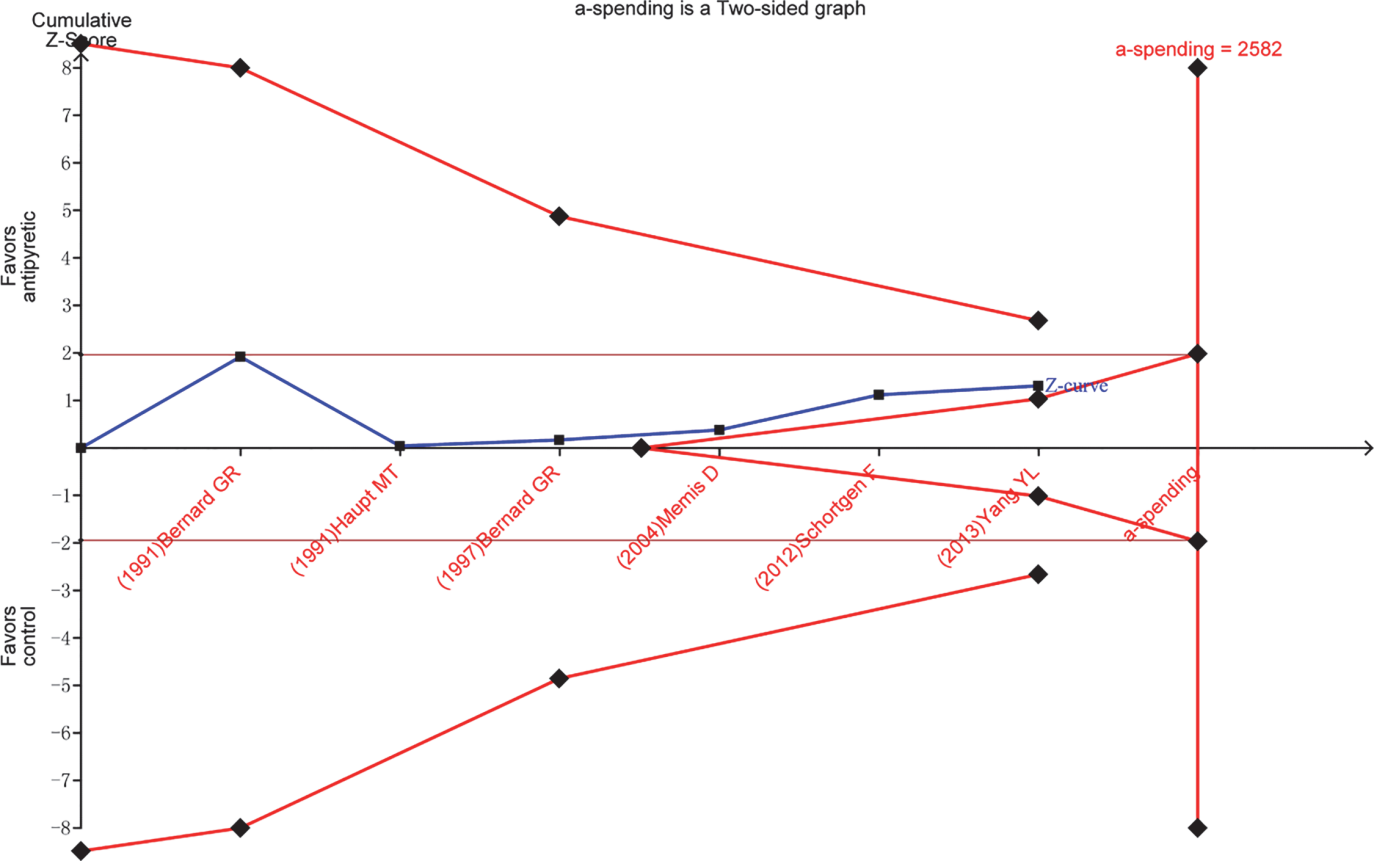
Meta-analysis with repeated non-superiority, non-inferiority and two-sided significance testing boundaries. The required information size (IS) was 2582 and our analysis has not yet reached half of the IS. The Z-curve did not cross the O’Brien-Fleming α-spending boundary or reach the futility, indicating that the non-significant result was probably due to lack of statistical power.

The six component trials included a total of 819 patients (Fig. 2). Only one study showed statistically beneficial effect of antipyretic therapy on mortality risk (OR: 0.44, 95% CI: 0.23–0.85), and other trials all showed neutral effect. There was significant heterogeneity among component trials as indicated by an I^2^ of 71.7% (p = 0.003). Overall, there was no beneficial effect of antipyretic therapy on mortality risk in patients with established sepsis (OR: 1.02, 95% CI: 0.50–2.05). The required information size (IS) was 2582 and our analysis has not yet reached half of the IS (Fig. 3). The Z-curve did not cross the O’Brien-Fleming α-spending boundary or reach the futility, indicating that the non-significant result was probably due to lack of statistical power.

### Publication bias

**Fig 4:**
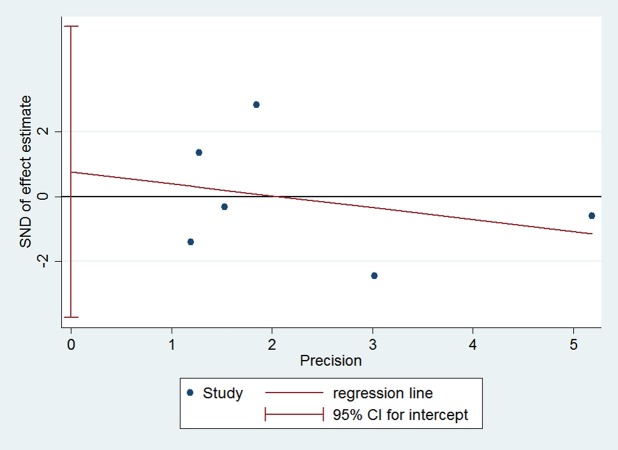
Publication bias was assessed by using Egger’s regression test. The standard normal deviate (SND) is regressed against the estimates precision. The intercept of the regression line is 0.76 (95% CI: -3.74–5.25), indicating no statistically significant publication bias.

Publication bias was assessed by using Egger’s regression test (Fig. 4). The standard normal deviate (SND) is regressed against the estimates precision. The intercept of the regression line is 0.76 (95% CI: -3.74–5.25), indicating no statistically significant publication bias.

### Subgroup analyses

**Fig 5:**
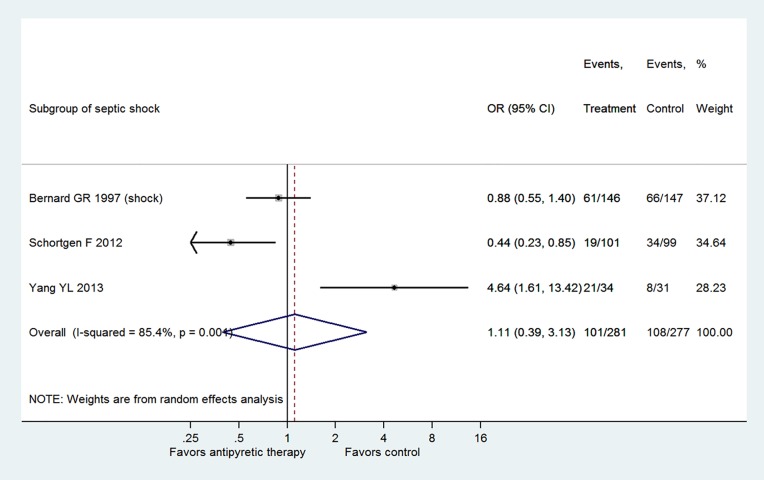
Subgroup analysis by restricting to patients with septic shock. Three trials fulfilled this criterion and the result showed there was no beneficial effect of antipyretic therapy on reducing mortality rate (OR: 1.11, 95% CI: 0.39–3.13).

**Fig 6:**
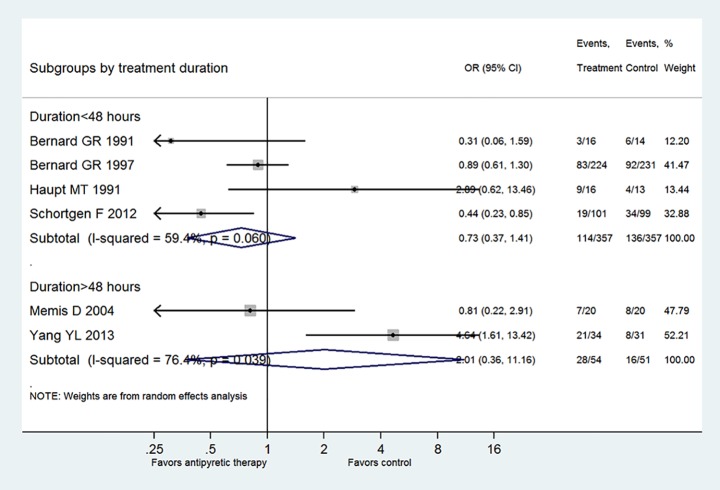
Subgroup analysis stratified by the duration of antipyretic therapy. The result showed that there was no treatment effect with antipyretic therapy in neither the subgroup with treatment<48 hours (OR: 0.73, 95% CI: 0.37–1.41) nor the subgroup with treatment>48 hours (OR: 2.01, 95% CI: 0.36–11.16).

**Fig 7:**
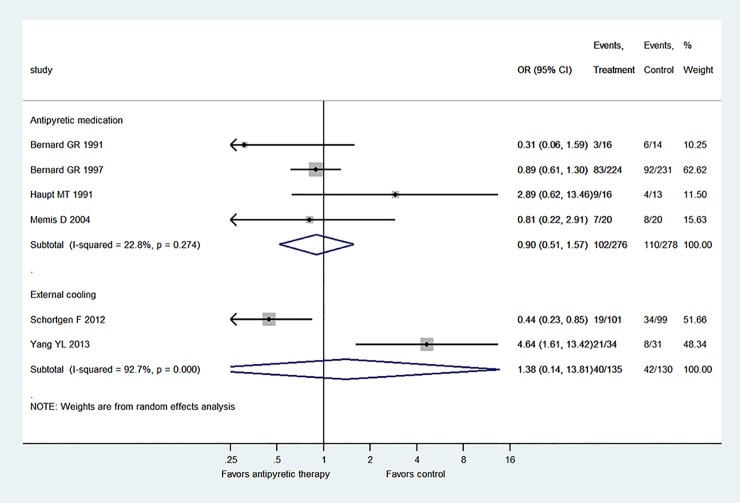
Subgroup analysis stratified by the mode of antipyretic therapy (antipyretic medication vs. external cooling). The result showed that there was no treatment effect with antipyretic therapy in neither the subgroup of antipyretic medication (OR: 0.90, 95% CI: 0.51–1.57) nor the subgroup of external cooling (OR: 1.38, 95% CI: 0.14–13.81).

Fig. 5 shows the subgroup analysis by restricting to patients with septic shock. Three trials fulfilled this criterion and the result showed there was no beneficial effect of antipyretic therapy on reducing mortality rate (OR: 1.11, 95% CI: 0.39–3.13). Also, Fig. 6 displays the subgroup analysis stratified by the duration of antipyretic therapy. The result showed that there was no treatment effect with antipyretic therapy in neither the subgroup with treatment<48 hours (OR: 0.73, 95% CI: 0.37–1.41) nor the subgroup with treatment>48 hours (OR: 2.01, 95% CI: 0.36–11.16). Fig. 7 shows the subgroup analysis stratified by the mode of antipyretic therapy (antipyretic medication vs. external cooling). The result showed that there was no treatment effect with antipyretic therapy in neither the subgroup of antipyretic medication (OR: 0.90, 95% CI: 0.51–1.57) nor the subgroup of external cooling (OR: 1.38, 95% CI: 0.14–13.81).

## Discussion

The study showed that antipyretic therapy was less likely to have beneficial effect for patients with established diagnosis of sepsis, and the result remained unchanged irrespective of the duration of treatment, mode of antipyretic therapy and shock status. However, the trial sequential analysis indicated that the null effect of the study was probably due to inadequate power as the Z-curve did not cross the futility line and the total sample size was less than half of the required information size. Therefore, future trials are still need to determine whether antipyretic therapy will be beneficial to septic patients.

One systematic review has been conducted by Niven DJ and coworkers,[8] in which critically ill patients with febrile were included as study population. Although this population included a large proportion of septic patient, it still differed from our study by including febrile patients that were not caused by infection. For instance, Niven’s systematic review included the study by Schulman CI that was conducted in trauma ICU and enrolled substantial number of patients of brain injury.[23] There is a large body of evidence suggesting potential beneficial effect of hyperthermia in inhibiting bacterial growth,[7,24,25] which has long been used as an argument against fever control in febrile patients with infection. This beneficial effect is not available in non-infectious febrile patients (e.g. fever caused by central nervous system), but the adverse effect of hyperthermia take predominance. The effect of antipyretic therapy for non-infectious febrile is less ambiguous,[26–28] but it is controversial in infectious febrile. Therefore, the present study aimed to solve this controversy. However, our study failed to identify any beneficial effect of antipyretic therapy in patients with sepsis, the same as that demonstrated by Niven DJ and coworkers.

The strength of the present study is that we employ trial sequential analysis, providing a better framework for the interpretation of the results. The meta-analysis included small number of population and small number of component trials, which is subject to random error and can cause spurious findings. Repeated update of meta-analysis involves a phenomenon commonly known as “multiplicity due to repeated significance testing”.[29] Required information size (number of patients) was calculated in our study and if this was reached, a reliable conclusion can be drawn from the meta-analysis. Unfortunately, the available evidence failed to reach half of the required information size. The solution to this problem is to use the α-spending function and trial sequential monitoring boundaries. The solution works to adjust the threshold for Z-value, allowing the type I error risk to restore the desired maximum risk.[30,31] The result of sequential trial analysis showed that the Z-curve did not cross the O’Brien-Fleming boundary, suggesting there was no evidence of benefits of the antipyretic therapy. Furthermore, the Z-curve did not cross the non-superiority boundary, indicating that the insignificance could be due to lack of statistical power.

In aggregate, our study failed to identify any beneficial effect of antipyretic therapy on ICU patients with established diagnosis of sepsis. Due to limited number of total participants, more studies are needed to make a conclusive and reliable analysis.

## Supporting Information

S1 Searching Searching strategy. (DOCX)
