# Supplementary material for: Antipyretic Therapy in Critically Ill Patients with Established Sepsis: A Trial Sequential Analysis
Source: PLoS One. 2015 Feb 24;10(2):e0117279. doi: 10.1371/journal.pone.0117279 (PMC4339198; doi:10.1371/journal.pone.0117279)
Supplement: S1 Searching — (DOCX) [file pone.0117279.s001.docx]

Pubmed

#1: **((sepsis[Title/Abstract]) OR septic[Title/Abstract]) OR infection[Title/Abstract]**

**#2:**  **(((antipyretic[Title/Abstract]) OR antipyretics[Title/Abstract]) OR cooling[Title/Abstract]) OR hypothermia[Title/Abstract]**

**#3: #1 AND #2**

#4: #3 Filters: **Clinical Trial**

**123 citations identified**

**Scopus**

**#1: TITLE-ABS-KEY( *septic*)OR TITLE-ABS-KEY( *sepsis*)OR TITLE-ABS-KEY( *infection*)**

**#2: TITLE-ABS-KEY( *antipyretic*) OR TITLE-ABS-KEY( *antipyretics*) OR TITLE-ABS-KEY( *cooling*) OR TITLE-ABS-KEY( *hypothermia*)**

**#3:   TITLE-ABS-KEY ( *random* )  OR  TITLE-ABS-KEY ( *randomized* )  OR  TITLE-ABS-KEY ( *randomization* )**

**#4: #1 AND #2 AND #3**

**#5: #4 AND LIMIT-TO ( DOCTYPE ,  "ar" )**

**387 citations identified**

**EBSCO**

**S1:** [**AB sepsis OR AB septic OR AB infection**](http://web.a.ebscohost.com/ehost/breadbox/search?term=AB%20sepsis%20OR%20AB%20septic%20OR%20AB%20infection&sid=bd1121aa-16d8-4b54-abf1-ce1b1bfa06a0%40sessionmgr4003&vid=4)

**S2:** [**AB antipyretic OR AB antipyretics OR AB cooling OR AB hypothermia**](http://web.a.ebscohost.com/ehost/breadbox/search?term=AB%20antipyretic%20OR%20AB%20antipyretics%20OR%20AB%20cooling%20OR%20AB%20hypothermia&sid=bd1121aa-16d8-4b54-abf1-ce1b1bfa06a0%40sessionmgr4003&vid=5)

**S3:** [**AB random OR AB randomized OR AB randomization**](http://web.a.ebscohost.com/ehost/breadbox/search?term=AB%20random%20OR%20AB%20randomized%20OR%20AB%20randomization&sid=bd1121aa-16d8-4b54-abf1-ce1b1bfa06a0%40sessionmgr4003&vid=6)

**S4: S1 AND S2 AND S3**

**35 citations identified**

**EMbase**

**#1: sepsis:ab OR septic:ab OR infection:ab**

**#2: antipyretic:ab OR antipyretics:ab OR hypothermia:ab OR cooling:ab**

**#3: #1 AND #2**

**#4: #3 AND 'clinical trial'/de**

**121 citations identified**
